# Genomic, Phylogenetic and Physiological Characterization of the PAH-Degrading Strain *Gordonia polyisoprenivorans* 135

**DOI:** 10.3390/biology13050339

**Published:** 2024-05-13

**Authors:** Ekaterina Frantsuzova, Alexander Bogun, Olga Kopylova, Anna Vetrova, Inna Solyanikova, Rostislav Streletskii, Yanina Delegan

**Affiliations:** 1 Institute of Biochemistry and Physiology of Microorganisms, Federal Research Center “Pushchino Scientific Center for Biological Research of Russian Academy of Sciences” (FRC PSCBR RAS), 142290 Pushchino, Moscow Region, Russia; frantsuzova.ee@gmail.com (E.F.); bogun62@mail.ru (A.B.); oa.kopylova01@gmail.com (O.K.); phdvetrova@gmail.com (A.V.); innap_solyan@yahoo.com (I.S.); 2 Pushchino Branch of Federal State Budgetary Educational Institution of Higher Education “Russian Biotechnology University (ROSBIOTECH)”, 142290 Pushchino, Moscow Region, Russia; 3 Regional Microbiological Center, Belgorod State University, 308015 Belgorod, Russia; 4 Laboratory of Ecological Soil Science, Faculty of Soil Science, Lomonosov Moscow State University, 119991 Moscow, Russia; streletskiyrostislav@mail.ru

**Keywords:** *Gordonia polyisoprenivorans*, genome analysis, pangenome, IGR analysis, biodegradation, polycyclic aromatic compounds

## Abstract

**Simple Summary:**

Polycyclic aromatic hydrocarbons are dangerous environmental pollutants and can be harmful to human health due to their carcinogenicity. The strain *Gordonia polyisoprenivorans* 135 is able to utilize such compounds and is therefore of interest for application in environmental biotechnology. We look inside the genome structure of this strain, analyze the genetic features of the catabolism of aromatics, and identify differences between the strain and its closest relatives at both the gene and intergenic levels. We also establish that the genome fragment carrying genes of aromatic catabolism is not characteristic of *Gordonia polyisoprenivorans*, but was most likely acquired externally from other related *Actinobacteria*. As a result, several interesting features of the evolutionary development of the genus *Gordonia* are revealed.

**Abstract:**

The strain *Gordonia polyisoprenivorans* 135 is able to utilize a wide range of aromatic compounds. The aim of this work was to study the features of genetic organization and biotechnological potential of the strain *G. polyisoprenivorans* 135 as a degrader of aromatic compounds. The study of the genome of the strain 135 and the pangenome of the *G. polyisoprenivorans* species revealed that some genes, presumably involved in PAH catabolism, are atypical for *Gordonia* and belong to the pangenome of *Actinobacteria*. Analyzing the intergenic regions of strain 135 alongside the “panIGRome” of *G. polyisoprenivorans* showed that some intergenic regions in strain 135 also differ from those located between the same pairs of genes in related strains. The strain *G. polyisoprenivorans* 135 in our work utilized naphthalene (degradation degree 39.43%) and grew actively on salicylate. At present, this is the only known strain of *G. polyisoprenivorans* with experimentally confirmed ability to utilize these compounds.

## 1. Introduction

Polycyclic aromatic hydrocarbons (PAHs) are common environmental pollutants. They appear in soils and aquatic ecosystems from fuel spills [1,2], as components of pesticides and PAH-containing media [3,4,5], in the atmosphere as a result of burning wood, coal, and automobile exhaust [6,7,8,9,10]. PAHs are toxic to humans and animals, and many are carcinogenic [11,12,13]. Removal of excessive amounts of PAHs from ground and aquatic ecosystems is an urgent problem of modern biotechnology.

Many representatives of the order *Mycobacteriales* of the class *Actinomycetia* (phylum *Actinomycetota*) are able to utilize PAHs. This ability has been observed in representatives of the families such as *Nocardiaceae* [14,15,16,17,18], *Dietziaceae* [19,20,21], *Mycobacteriaceae* [22,23] and others. *Rhodococcus* strains are more commonly known for their ability to degrade PAHs compared to other members of this taxonomic group [24,25,26]. For example, *Rhodococcus* bacteria in work [27] utilized 43% of PAH mixture (naphthalene, acenaphthene, anthracene, phenanthrene, benzo[a]anthracene, and benzo[a]pyrene, total PAH content 1 g/kg) in soil within 213 days. The strain *Rhodococcus* sp. A2-3 [28] utilized 89% of fluorene (initial concentration 0.4 g/L) in 7 days.

*Gordonia* strains (family *Gordoniaceae*, order *Mycobacteriales*) are not currently used in environmental biotechnology as much as *Rhodococcus*. However, the plasticity of genomes and metabolic flexibility of representatives of the genus *Gordonia* make them interesting for both fundamental studies and practical applications. *Gordonia* strains are mainly known as degraders of alkanes [29,30,31], but among them there are also degraders of thiophenes [26,32], phthalates [33,34,35] and steroids [36,37,38].

The ability of *Gordonia* strains to utilize sulfur-containing PAH derivatives has been reported repeatedly. Thus, the strain *Gordonia* sp. IITR100, using a chain of sequential reactions, converts benzonaphthothiophene via benzo[b]naphtho-[2,1-d] thiophene 11,11-dioxide to 2-phenyl-naphthalen-1-ol [39] and thianthrene via thianthrene 5,5-dioxide to o-hydroxyphenyl phenylsulfone [40]. The utilization of benzothiophenes and dibenzothiophenes is a common ability of members of the genus. Previously, it was believed that this process required the *dsz* gene cluster for control [41,42]. However, we later demonstrated that growth on thiophenes is also possible in *Gordonia* strains lacking the *dsz* cluster [43]. It should be noted that the conversion process of sulfur-containing aromatic compounds affects only the ring with sulfur atoms, as a consequence of which sulfur is extracted from the compound structure. Other aromatic rings remain unavailable for utilization by *Gordonia* strains; therefore, the ability to desulfurize sulfur-containing aromatic compounds does not necessarily imply the ability to cleave hydrocarbon aromatic rings.

Regarding the degradation of unsubstituted PAHs, there are only a few reports of such ability in representatives of *Gordonia*. Young et al. [44] observed the growth of *Gordonia alkanivorans* strain CC-JG39, isolated from oil-contaminated sludge in Taiwan, on naphthalene at a concentration of 1 g/L. *G. alkanivorans* strain H19 [45] did not grow on naphthalene and phenanthrene, but did grow on pyrene. At the same time, none of the nine strains we investigated as part of our study on the *G. alkanivorans* pangenome [46] utilized aromatic compounds.

Kurniati et al. [47] observed the ability to degrade pyrene in *Gordonia cholesterolivorans* strain AMP 10. The strain *G. iterans* Co17 utilized naphthalene and anthracene in oil with degradation rates of 55.3% and 63.2%, respectively. Hu et al. [48] isolated three strains of *Gordonia* sp. from PAHs-contaminated soil in China. The strains demonstrated utilization abilities for various PAHs, including pyrene, benzo[a]pyrene, anthracene, naphthalene, phenanthrene, and fluoranthene.

Since the ability to utilize PAHs is rare in *Gordonia*, unlike a similar ability observed, for example, in *Rhodococcus*, our interest was piqued by the discovery of a PAH-degrading *Gordonia* strain, *Gordonia polyisoprenivorans* 135, isolated in 1998 from soil contaminated with oil, diesel fuel, and chlorinated aromatic compounds (Samara, Russia) [49]. The aim of this work was to study the features of genetic organization and biotechnological potential of *G. polyisoprenivorans* strain 135 as a degrader of naphthalene and its derivatives.

## 2. Materials and Methods

### 2.1. Bacterial Strain, Media and Cultivation Conditions

The strain *Gordonia polyisoprenivorans* 135 is maintained in the Laboratory of Microbial Physiology of IBPM RAS (Pushchino, Moscow region, Russia). To maintain the strain’s properties as a degrader of aromatic compounds, culture reseeding was performed every 2 months on agarized mineral medium CP1 with salicylate (0.1 g/L).

The CP1 mineral medium [50] had the following composition: (g/L): Na_2_HPO_4_, 0.7; KH_2_PO_4_, 0.5; NH_4_NO_3_, 0.75; MgSO_4_ × 7H_2_O, 0.2; MnSO_4_, 0.001; FeSO_4_, 0.02. Lysogeny broth (LB) medium consisting of (per liter of distilled water) 10 g of tryptone, 5 g of yeast extract, 5 g of NaCl, and 15 g of agar (Panreac, Spain) was used to test the purity of the bacterial culture and to obtain individual colonies.

The strain was cultured at 28 °C on CP1 medium supplemented with the following carbon and energy sources: naphthalene (2 g/L), benzoate (1 g/L), catechol (0.1 g/L), salicylate (0.1 g/L) on an orbital shaker at 180 rpm. All the reagents were obtained from Sigma-Aldrich (USA). Inoculum was prepared according to the method described in [51], Section 2.3.

### 2.2. Bioinformatic Analysis of the Genome of G. polyisoprenivorans 135

The protocol for sequencing and assembly of the genome of *G. polyisoprenivorans* strain 135 is described in [52]. To assess the quality of the genome, CheckM v. 1.2.2 [53] was used. The genome contains a total of 5364 genes, of which 5303 are coding sequences (CDSs). Of the 5303 CDSs, 5168 were CDSs with protein and 135 were pseudogenes. The genome contains a total of 61 RNA genes, of which 49 tRNAs, 3 ncRNAs, and 9 rRNAs. The genomic data for *Gordonia polyisoprenivorans* strain 135 are available in the GenBank database under accession number CP116236.1 (BioProject PRJNA923796, BioSample SAMN32738803).

The whole-genome tree was built using the TYGS web service [https://tygs.dsmz.de/ (accessed 23 November 2023)] from Genome BLAST Distance Phylogeny (GBDP) distances using “greedy-with-trimming” algorithm. GBDP defines distances between pairs of fully or partially sequenced genomes. The algorithm “greedy-with-trimming” involves removing the overlapping parts of high-scoring segment pairs in either genome [54].

The ANI value was calculated using the EzBioCloud ANI Calculator [55]. DNA-DNA hybridization (DDH) was calculated using the Genome-to-Genome Distance Calculator (GGDC) [54].

The alignment maps were constructed using the program Mauve ver. 2.4.0, (21 December 2014) [56]. PanExplorer software [57] was used to analyze the pangenome and search for unique genes. The genome maps were constructed using Circos (for pangenome analysis) [58], Proksee (accessed on 30 September 2023) [59] and MG2C [60] services. Functional annotation of the genome was performed using KEGG [61]. MGE search was performed using Mobile-OG db [62]. Alien_Hunter [63] was used to detect horizontally transferred regions in the genome. For specific gene finding BLAST search was carried out using “Nucleotide collection (nr/nt)” and “Whole-genome shotgun contigs (WGS)” databases.

Metabolic pathways were drawn using the program ChemDraw Ultra ver. 12.0.2.1076.

To analyze the noncoding pangenome, we used Piggy [64] and Roary [65] with the assistance of GNU Parallel [66]. Rho-dependent terminators were identified using RhoTermPredict [67], while Rho-independent terminators were identified using iTerm-PseKNC [68] and Arnold [69]. Prediction of noncoding RNAs in the genome of the strain was conducted using StructRNAfinder [70] and RNAcentral (https://rnacentral.org/, release 23, accessed on 15 March 2024).

Statistical processing was carried out using R version 3.6.3 (29 February 2020), and visualization of the results was performed using ggplot2 [71].

### 2.3. Cultivation of G. Polyisoprenivorans Strain 135 and Evaluation of Its Efficiency as a Degrader of Aromatic Compounds

The ability of strain *G. polyisoprenivorans* 135 to degrade aromatic compounds was tested in 100 mL of mineral medium CP1 [50] containing 2 g/L naphthalene or 0.1 g/L salicylate for 7 days at 27 °C. For inoculum preparation, the strain was grown for 24 h in mineral medium supplemented with 10% *w*/*w* potassium acetate as a carbon and energy source. The grown biomass was precipitated and washed with phosphate-buffered saline (PBS) solution. The resulting biomass was then resuspended in PBS solution to a concentration of 1 × 10^8^ CFU/mL using a McFarland turbidity standard. The inoculum was introduced into the experimental system so that the inoculum dose did not exceed 1 × 10^6^ CFU/mL. Cell numbers were estimated by counting colonies grown on LB agar medium at standard serial dilutions during 7 days of cultivation at 27 °C. All the experiments were performed in three independent biological replicates.

### 2.4. Measurement of Naphthalene Degradation Degree in the Medium

Naphthalene was extracted from the growth medium by mixing with dichloromethane (1:2, *v*/*v*). Gas chromatography (Agilent 6890, Agilent Technologies, Santa Clara, CA, USA) with a flame ionization detector was used to estimate the concentration of naphthalene in 100-fold diluted extracts. The equipment of the Collective Use Centre of the Faculty of Soil Science and of the Lomonosov Moscow State University was used. A DB-1 column (30 m × 0.25 mm id, 0.25 μm) was used as the chromatographic column. The oven temperature was increased by 15 °C per minute. The initial temperature was 40 °C.

The maximum temperature was 300 °C, held for 5 min. Mode without flow splitting (in non-splitting mode) was chosen. Injection volume was 1 µL. Helium was used as a makeup gas. Helium flow rate was 1 mL/min.

The correlation coefficient was found to be 0.97. The ANOVA was *p* = 0.05.

The following formula was used to calculate the degree of naphthalene biodegradation (N):

N = (N_0_ − Ni)/N_0_ × 100 [%],

where N_0_ is the concentration of naphthalene in the experiment without microorganisms after 7 days; Ni is the concentration of naphthalene in the experiment with microorganisms after 7 days.

### 2.5. Detection of Naphthalene Metabolites

Salicylate and catechol were measured using a high-performance liquid chromatography (HPLC) system (Agilent 1260, Agilent Technologies, Santa Clara, CA, USA) equipped with the UV-detector. The wavelengths were as follows: catechol, 280 nm; salicylate, 300 nm. A Synergi Hydro-RP chromatographic column (150 × 4.6 mm id, 4 µm) was used. The temperature of the column thermostat was 25 °C; the volume of the injected sample was 10 µL. Eluents: A, 90% water: 5% acetonitrile: 5% 0.1% trifluoroacetic acid; B, 95% acetonitrile: 5% 0.1% trifluoroacetic acid. Flow rate, 0.75 mL/min. Elution in gradient mode: 0 min, 5%; 15 min, 15%; 22.5 min, 40%; 25 min, 40%; 25.5 min, 95%; 30 min, 95%.

The presence of naphthalene metabolites was determined on the third, fifth and seventh day of the experiment.

## 3. Results and Discussion

### 3.1. Cultural and Morphological Characteristics of the Strain G. polyisoprenivorans 135

The strain *G. polyisoprenivorans* 135 forms rough colonies that are light beige to cream-colored on agarized media (Figure S1).

**Figure 1:**
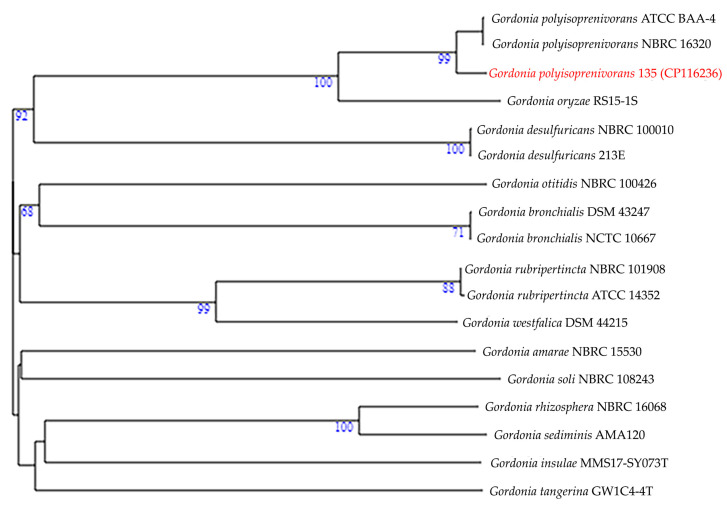
Whole-genome tree demonstrating the position of strain 135 (red) within the species *G. polyisoprenivorans* and within the genus *Gordonia* in general.

Strain 135 was originally identified as *Rhodococcus rhodnii* based on culture and morphological features and a number of biochemical tests [72]. Misidentification of *Gordonia* strains as *Rhodococcus* is a common phenomenon of the past when the methods in place at the time did not distinguish between the two related genera [73]. By ANI value (98.68%) and DDH value (88.40%), the strain is reliably included in the species *G. polyisoprenivorans* and clusters with the type *Gordonia polyisoprenivorans* strain on the phylogenetic tree (Figure 1).

At the moment (February 2024), the Genbank database contains 11 assemblies of *Gordonia polyisoprenivorans* genomes. Three of them belong to the type strain of *G. polyisoprenivorans*, which is maintained in different culture collections (Table 1).

When identifying strain 135, we used the ATCC BAA-14 assembly (JAAXPC000000000000.1) as a reference due to its superior quality in terms of coverage and number of contigs. In addition to strain 135, the genomes of three *G. polyisoprenivorans* strains are represented as complete polished assembly, while one strain (HW436) is represented as scaffold (Table 2).

Of the completely assembled genomes, strains *G. polyisoprenivorans* C and HW436 are the closest to strain 135. The strain *Gordonia* HW436 was sequenced and studied by Woo et al. [74] as a lignin degrader. Meanwhile, the strain *G. polyisoprenivorans* C is known as a carbamazepine degrader [75]. Carbamazepine, a widely used medication for epilepsy treatment, is a nitro-substituted three-ring aromatic compound, with the seven-carbon middle ring containing a nitrogen atom [76,77].

### 3.2. Horizontal Gene Transfer (HGT) Regions in the Genome of G. polyisoprenivorans Strain 135

According to Vos et al. [78], the acquisition of genetic elements is a key driver of bacterial evolutionary development. A clear example of this is the transfer of catabolic plasmids, as well as mobile genetic elements carrying specific metabolic pathways that integrate into chromosomes. As a result of this process, even distantly related taxa give rise to microorganisms with expanded catabolic properties, as well as the formation of an extensive gene pool whose exact source cannot be determined.

We identified 74 horizontal gene transfer (HGT) regions of total length 498 kbp scattered throughout the genome of the strain. These sites can be arranged in groups or single, with the longest one being 27.5 kbp (Figure S2). HGT regions are mainly home to repeat elements, transposases, IS elements and hypothetical proteins, although some catabolic genes, particularly those for the degradation of aromatic compounds, are also present (see below).

Horizontal transfer in *Actinobacteria*, especially soil bacteria, is a common phenomenon [79,80], but few examples of this event are specifically known in *Gordonia* strains with respect to catabolic genes. Jung et al. [81] suggested that the plasmid pGKT2 bearing genes of hexahydro-1,3,3-trinitro-1,3,5-triazine (RDX) degradation (gene locus *xpl*AB) was obtained by the strain *Gordonia* sp. KTR9 during horizontal transfer. The authors demonstrated transfer of the plasmid within the genus (to the recipient strain *Gordonia polyisoprenivorans*), and successful intergeneric transfer to the strains *Rhodococcus jostii* RHA1 and *Nocardia* sp. TW2. Consequently, the recipient strains acquired the ability to utilize RDX.

Heine et al. [82] suggested that the ability to produce glutathione was acquired by *G. rubripertincta* strain CWB2 during the uptake of plasmids bearing genes for isoprene degradation. This assumption was confirmed by a different GC content region (in contrast to the main chromosome) and the presence of several mobile elements in close proximity to the genes for isoprene degradation. The authors noted a high percentage of similarity of this region with similar parts of the genomes of *R. opacus* strain 1CP and *R. jostii* strain RHA1.

### 3.3. Pangenome Analysis of Strain 135 and Its Closest Relatives

#### 3.3.1. The Pangenome of Coding Regions

**Figure 2:**
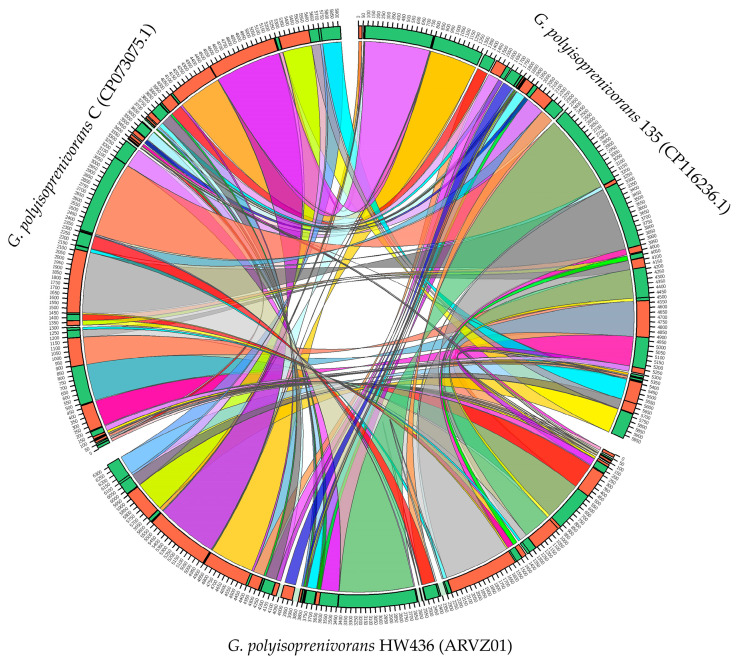
Circos visualization of locally collinear blocks identified between chromosomes of *Gordonia polyisoprenivorans* 135, C and HW436.

The chromosome structures of *G. polyisoprenivorans* strains 135, C, and HW436 have some differences in the arrangement of elements, but the genomes are generally similar (Figure 2). We found 70,749 SNPs between genomes 135 and HW436, and 79,323 SNPs between genomes 135 and C, which are evenly distributed throughout the genome. Single-nucleotide substitutions in the genome of strain 135 relative to the genomes of related strains account for 1.1–1.3% of the total genome length.

The *G. polyisoprenivorans* pangenome was analyzed using a dataset comprising three strains: *G. polyisoprenivorans* 135 and *G. polyisoprenivorans* C (CP073075.1) which are closely related, and *G. polyisoprenivorans* VH2 (CP003119.1), which is phylogenetically distinct from this pair. We aimed to identify differences not only between the closest relatives but also to observe which genes are unique to a strain that stands phylogenetically apart from them.

**Figure 3:**
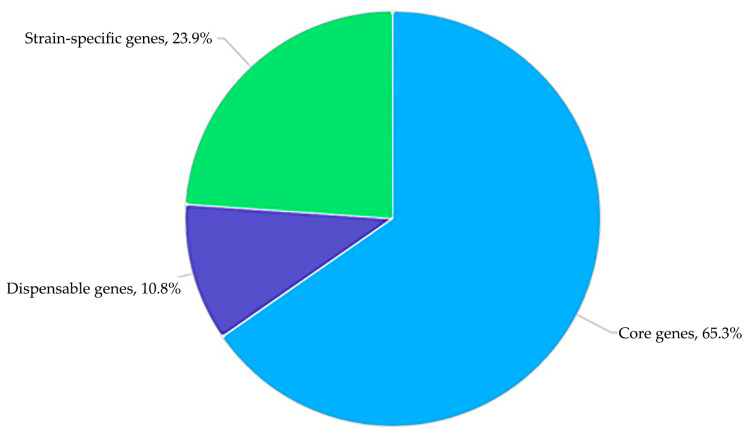
Distribution of core-genome and accessory genome between *G. polyisoprenivorans* 135, *G. polyisoprenivorans* C and *G. polyisoprenivorans* VH2.

The pangenome of the three strains (Figure 3 and Figure S3) consists of 6259 genes, with 4089 classified as core genes. Dispensable genes, defined as those absent in at least one of the strains [83], comprise 674 genes (10.8%).

The distribution of COG functional categories in the strains is similar (Figure S4). Among the genes unique to strain 135 (Table S1) in pangenome, it is interesting to note those related to the degradation of aromatic compounds (see below).

#### 3.3.2. The Pangenome of Non-Coding Regions

The concept of the pangenome is primarily focused on protein-coding open reading frames (ORFs) [84,85]. However, such an approach automatically excludes non-coding regions, which constitute up to 15% of the total genome length [86,87]. Non-coding regions include structurally important elements of genomes such as promoters, terminators, virulence factors, and non-coding RNAs [88,89]. Acquisition or, conversely, exclusion of these regions from the genome can significantly influence the phenotype.

We analyzed the diversity of non-coding regions in the genome of the strain 135 and the pangenome of *G. polyisoprenivorans*. For analysis, in addition to strain 135, we included four strains of *G. polyisoprenivorans* with completely assembled genomes: HW436 (NZ_ARVZ01000001.1), C (CP073075.1), R9 (CP072203.1), and VH2 (CP003119.1).

There are two perspectives on the transfer of intergenic (regulatory) regions. The first suggests that the connection between the coding region and the upstream-located non-coding region prevents them from moving independently of each other [90]. The second allows for the possibility of the separate movement of regulatory regions, a phenomenon known as horizontal regulatory transfer (HRT) [91,92]. Such regulatory regions are referred to as “switched”. During the analysis of the non-coding pangenome (Thorpe et al. [64] proposed the term “panIGRome”), in cases where different intergenic regions corresponded to the same downstream gene in genomes of different strains, we considered such intergenic regions to be switched.

Earlier studies on strains of *S. aureus* [64] and *E. coli* [92] have shown that genes with switched upstream intergenic regions exhibit a higher level of expression compared to genes with “native” (i.e., non-switched) intergenic regions.

1834 intergenic regions of 100–1000 bp in length were detected in the genome of the strain 135, 71 of them were considered as switched, meaning they differ in their sequences from the sequences lying between pairs of analogous genes in other strains of *G. polyisoprenivorans* (Figure S5). The IGR (1730231-1730449) in the region containing genes involved in the catabolism of aromatic compounds measure 218 bp. This IGR partially overlaps with sequences of non-coding RNAs such as the *Actinomyces denticolens* FMN riboswitch (RFN element) and the *Ruminococcus* sp. CAG:9-related_41_34 FMN riboswitch (RFN element).

#### 3.3.3. Search for Non-Coding RNAs in the Genome of *G. polyisoprenivorans* Strain 135

In the genome of the strain, we identified 140 sequences corresponding to families of non-coding RNAs (ncRNAs) from the Rfam database. Further inspection showed that 34 of them corresponded to ncRNAs predicted for *Actinobacteria* of the genera *Gordonia*, *Rhodococcus*, and *Mycobacterium*. We compared the sequences and localization of elements, potentially corresponding to ncRNAs, with the results obtained from the search for intergenic regions (IGRs).

We discovered a switched non-coding region upstream of the gene encoding 4-hydroxybenzoate 3-monooxygenase. The product of this gene controls the hydroxylation reaction of the aromatic compound 4-hydroxybenzoate [93]. The intergenic region, 197 nucleotides in length, is located between the HTH-type transcriptional regulator *kip*R and 4-hydroxybenzoate 3-monooxygenase *pra*I. We found two terminators in the region, but sequences corresponding to ncRNAs were absent.

A region 65 nucleotides long, located at coordinates 2407663–2407727, was identified as *ykk*C-III. This ncRNA is not only found in *Gordonia* but also in other *Actinobacteria*, and according to several studies [94,95], it is involved in regulating the removal of guanidine from cells. The Rfam database provides a broader characterization: according to Rfam, *ykk*C regulates the function of efflux pumps and participates in the removal of toxic compounds, including xenobiotics. We also detected several riboswitches, a 6C RNA sequence with the function of stress response [96], and an F6 sRNA sequence. According to [97], F6 sRNA modifies expression of chaperonins and is induced in starvation conditions.

### 3.4. Growth Characteristics of Strain 135 on Aromatic Compounds

Some *Gordonia* strains are known to be able to degrade PAHs [98,99,100]. The strain *Gordonia* sp. Q8 was capable of degrading not only individual PAHs (naphthalene and pyrene) but also a mixture of naphthalene, phenanthrene, anthracene and pyrene in mineral medium [99]. The strain *Gordonia* sp. Q8 was capable of degrading naphthalene at a concentration of 0.5 g/L in 3 days by 70% (net of abiotic loss of PAHs). In our results, the degradation rate of naphthalene by the strain 135 was 39.43 ± 5.25% in 5 days relative to the control system without microorganisms. However, the initial level of naphthalene in the system was 2 g/L, which was 4 times higher compared to the data of the article [99]. The results indicate a high biodegradative potential of the strain 135 with respect to naphthalene.

**Figure 4:**
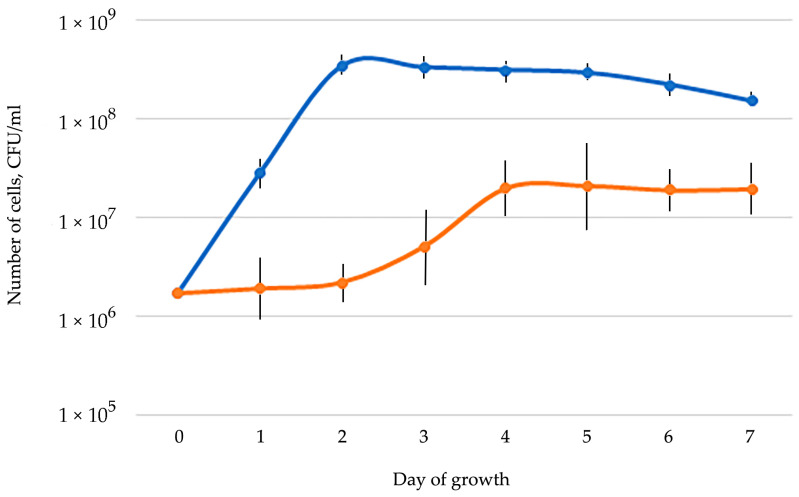
Growth curve of strain 135 on mineral medium with salicylate (blue) and naphthalene (orange).

In many prokaryotes, the ability to degrade naphthalene is realized through formation and further consumption of salicylate [101]. The strain *G. polyisoprenivorans* 135 is capable of growth on salicylate. Moreover, the culture reached the stationary phase on salicylate (0.1 g/L) in 2 days, while on naphthalene this period was 4 days (Figure 4).

Jacques et al. [102] reported a strain of *G. polyisoprenivorans* capable of utilizing pyrene, anthracene, phenanthrene, but not naphthalene or salicylate. The strain *G. polyisoprenivorans* strain 135 actively utilizes salicylate, a key metabolite of the naphthalene degradation pathway. At present, this is the only known strain of *G. polyisoprenivorans* with experimentally confirmed ability to utilize naphthalene and salicylate.

### 3.5. Assumptions about the Organization of Pathways and Mechanisms of Naphthalene Degradation in the Strain Gordonia Polyisoprenivorans 135

The genetic organization of the PAH catabolism pathway in *Gordonia* was initially described by Lin et al. [103]. The operon includes genes for rubredoxin, GntR-like and XylR-like regulators, the large (*nar*Aa) and small (*nar*Ab) subunits of naphthalene dioxygenase, naphthalene dihydrodiol dehydrogenase *nar*B, hydratase aldolase *nar*C, and a gene designated *orf*7 with unknown function. It is interesting to note that the *nar* operon in strain *Gordonia* sp. CC-NAPH129-6 [103] appears to have been acquired through horizontal transfer, as it is located on a 97 kb plasmid. Comparison of the sequences of its elements and phylogenetic markers (16S rRNA, *gyr*B) led the authors to suggest that *Rhodococcus* strain could be its donor.

The degradation of naphthalene In *Actinobacteria* mainly proceeds through salicylate [104,105]. However, there are strains where the naphthalene degradation pathway does not involve salicylate formation, such as *Rhodococcus opacus* strain M213 [16]. The authors noted that the strain did not utilize salicylate as a carbon and energy source.

Salicylate can be converted to catechol by the action of salicylate 1-hydroxylase [106,107] or gentisate by the action of salicylate 5-hydroxylase [104]. There are also reports of the conversion of salicylate to 2-oxohepta-3,5-dienedioic acid by the action of salicylate 1,2-dioxygenase. Such a reaction has been reported for the Gram-negative strain *Pseudaminobacter salicylatoxidans* [108], but there are no examples of this reaction in *Actinobacteria*.

In our work, the strain *G. polyisoprenivorans* 135 grew actively on salicylate and catechol and did not grow on gentisate; therefore, we hypothesize that the pathway of naphthalene degradation in strain 135 includes the stages of transformation of naphthalene to salicylate and then salicylate to catechol. HPLC-analysis showed no accumulation of metabolites, so we assume that all formed intermediates are immediately utilized in subsequent reactions.

Interestingly, despite the experimentally confirmed ability of strain 135 to utilize naphthalene and salicylate, we found no *nar* genes previously detected in *Gordonia* [103]. Analyzing the genomes of other *Gordonia* strains from the Genbank database, we found *nar* sequences in the genomes of strains *G. namibiensis* NBRC 108,229 (BAHE01000003.1), *G. metallireducens* tSed Te1 (JAJQJP010000036.1), *G. rubripertincta* BP295 (JAFFGU010000009.1), and three strains of *G. paraffinivorans*. Based on gene sequences, we assume that *nar* genes in *Gordonia* were acquired from *Rhodococcus* and *Streptomyces*.

We also did not find salicylate hydroxylase genes in the genome of strain 135. In addition, it is worth mentioning that salicylate hydroxylases are not common in *Gordonia* strains. According to BLAST search results using the WGS and nr/nt databases as references, these genes were found only in strains *Gordonia* sp. PDNC005 (CP070351.1), *Gordonia* sp. MMO (JBBCWK010000002.1), *Gordonia malaquae* MMO-152 (JBBCUB010000003.1), *Gordonia spumicola* NBRC 107,696 (BJOV01000003.1), and *Gordonia liuliyuniae* HY366 (JAKKOR010000002.1). These genes are mostly found in *Rhodococcus* strains, which, as in the case of the *nar* operon, suggests acquisition by horizontal transfer. Conversely, despite the presence of the gentisate 1,2-dioxygenase gene in the genome of strain *G. polyisoprenivorans* 135, the strain is unable to utilize this compound.

We identified genes responsible for *ortho*- and *meta*-pathways of catechol degradation in the genome of strain *G. polyisoprenivorans* 135.

Instead of *nar* genes, during genome annotation and pangenome analysis we identified six genes potentially involved in the catabolism of aromatic compounds in the genome of strain 135 (Table 3).

**Figure 5:**
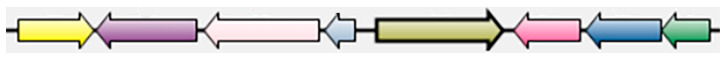
The structure of the region containing genes involved in the catabolism of aromatic compounds in strain 135. Gene products from left to right: IclR family transcriptional regulator, extradiol ring-cleavage dioxygenase WCB38962.1, FAD-dependent oxidoreductase, non-heme iron oxygenase ferredoxin subunit WCB38964.1, aromatic ring-hydroxylating dioxygenase subunit alpha WCB38965.1, dienelactone hydrolase family protein, SDR family oxidoreductase, aromatic-ring-hydroxylating dioxygenase subunit beta WCB38968.1.

The first 4 genes belong to the same region (Figure 5).

The genes encoding enzymes involved in opening and cleavage of aromatic rings in the genome of strain 135 can be divided into the following categories:

1. aromatic ring-hydroxylating dioxygenase group.

These genes encode enzymes that convert aromatic structures to cis-diols [109]. We found 4 such genes in the genome (Table 3). Comparison of the amino acid sequences of these genes did not reveal any relatedness between them.

2. extradiol ring-cleavage dioxygenase group.

Unlike intradiol enzymes, which use non-haem Fe(III) to open an aromatic ring via the ortho-pathway, extradiol enzymes use non-haem Fe(II) and cleave aromatic rings via the meta-pathway (between a hydroxylated carbon atom and an adjacent non-hydroxylated carbon atom) [110,111]. We identified three genes encoding extradiol ring-cleavage dioxygenase in the genome of strain 135.

One of the extradiol dioxygenases, iron-dependent extradiol dioxygenase HsaC, is involved in the degradation of steroid compounds. The enzyme encoded by the *hsa*C gene catalyzes the meta-cleavage of 3,4-dihydroxy-9,10-seconandrost-1,3,510-triene-9,17-dione (3,4-DHSA) to produce 4,5-9,10-diseco-3-hydroxy-5,9,17-trioxoandrosta-110,2-diene-4-oic acid (4,9-DSHA) [38]. Genes related to steroid catabolism are commonly found in various species of *Gordonia* genus. Zhang et al. [38] studied the organization of steroid catabolism genes in *Gordonia neofelifaecis* strain NRRL B-59395, which is a type strain of the *G. neofellifaecis* species. The ability of strain NRRL B-59395 to transform steroid compounds has been experimentally confirmed [112]. Additionally, in the work of Lee et al. [113] the strain *Gordonia* sp. JH63 was found to possess genes of cholesterol catabolism.

The extradiol ring-cleavage dioxygenase (WCB38962.1) and aromatic ring-hydroxylating dioxygenase subunit alpha (WCB38965.1) genes are not commonly found in the gene pool of *G. polyisoprenivorans.* Based on the sequences of these genes, there is no specific bacterial genus in which they would be most frequently found, indicating that they do not have a definite source. Sequences related to these genes are found in bacteria of the genera such as *Streptomyces*, *Rhodococcus*, *Mycobacterium*, and *Mycolicibacterium* (Table S2). These genes are located at the HGT site of 7501 bp in the coordinates 1,727,500–1,735,000.

Thus, summarizing the experimental data and bioinformatic analysis, we assume the following mechanism of naphthalene utilization by the strain *G. polyisoprenivorans* 135. The process is carried out through the formation of salicylate, but without its accumulation in the culture medium. The question of genetic control of salicylate conversion to catechol is still open; salicylate 1-hydroxylase is absent in the genome of the strain. According to Roy and Kastner [108], it is possible that extradiol dioxygenases may be involved in the salicylate conversion, the reaction results in the formation of 2,7-dioxo-3-hydroxyhepta-3,5-dienoic acid. However, there are no examples of this reaction in *Actinobacteria*, so we assume that the involvement of extradiol ring-cleavage dioxygenases is limited to catechol conversion processes. The genes of *ortho*- and *meta*-pathways of catechol conversion are represented in the genome.

## 4. Conclusions

The strain *Gordonia polyisoprenivorans* 135 possesses an interesting set of genes for the catabolism of aromatic compounds. Interestingly, some of these genes are not typical genes of *Gordonia*, but belong to the cloud-pangenome of *Actinobacteria*. Several genes of PAH catabolism are located within horizontally transferred region.

Some sequences of intergenic regions in the genome of strain 135 differ from the IGRs located between the same gene pairs in the genomes of related *G. polyisoprenivorans* strains. Additionally, we made several predictions of ncRNAs that could potentially be involved in catabolism of pollutants by strain 135. These findings offer deeper insights into the genetic organization of PAH catabolism in *Gordonia* strains.

The strain *G. polyisoprenivorans* 135 in our work utilized naphthalene and grew actively on salicylate. At present, this is the only known strain of *G. polyisoprenivorans* with experimentally confirmed ability to utilize these compounds.

## Figures and Tables

**Table 1:** Comparison of genome assemblies of the type strain of G. polyisoprenivorans maintained in different collections.

	NBRC 16320	JCM 10675	ATCC BAA-14
Genbank acc. Number	BAEI00000000.1	BBGD00000000.1	JAAXPC000000000.1
Submitted	01-DEC-2011	18-JUN-2014	06-APR-2020
Sequencing technology	Roche 454	Ion PGM	Illumina NovaSeq
Coverage	16x	35x	206.9x
GC%	66.90	65.60	66.90
Contigs number	113	2876	54
Total length, bp	6,285,478	4,974,030	6,287,369

**Table 2:** *G. polyisoprenivorans* strains from the Genbank database with completely assembled genomes. The type strain *G. polyisoprenivorans* ATCC BAA-14 was used as reference.

Strain Name	135	C	VH2	R9	HW436
Genbank acc. Number	CP116236.1	CP073075.1	CP003119.1, CP003120.1	CP072203.1	ARVZ01
Total length, Mb	5.99	5.93	5.84	6.03	6.33
Size of plasmid(s), kb	-	-	174	-	-
CDS number	5168	5147	5100	5286	5509
ANI value with type strain, %	98.68	98.42	98.19	94.14	98.64
DDH value with type strain, %	88.30	87.90	79.40	72.60	90.40
ANI value with the strain 135, %	-	98.39	98.01	93.94	98.49
DDH value with the strain 135, %	-	84.90	81.10	73.80	83.50

**Table 3:** The genes in the genome of strain 135 involved in the degradation of aromatic compounds. The genes in rows 2–4 are present only in the genome of strain 135, but not in the genomes of strains *G. polyisoprenivorans* R9, C and VH2.

	Gene Accession Number	Position in the Genome	Product Name
1	WCB38965.1	1730449..1731774	aromatic ring-hydroxylating dioxygenase subunit alpha
2	WCB38968.1	1733415..1733933	aromatic-ring-hydroxylating dioxygenase subunit beta
3	WCB38962.1	1727528..1728595	extradiol ring-cleavage dioxygenase
4	WCB38964.1	1729905..1730231	non-heme iron oxygenase ferredoxin subunit
5	WCB37763.1	226176..227519	aromatic ring-hydroxylating dioxygenase subunit alpha
6	WCB38820.1	1559611..1560738	aromatic ring-hydroxylating dioxygenase subunit alpha

## Data Availability

The genomic data are available in the GenBank database under accession number CP116236.1 (BioProject PRJNA923796, BioSample SAMN32738803).

## Supplementary Materials

The following supporting information can be downloaded at: https://www.mdpi.com/article/10.3390/biology13050339/s1, Figure S1: Colony appearance of the strain *Gordonia polyisoprenivorans* 135; Figure S2: Circular genomic map of *G. polyisoprenivorans* 135 chromosome; Figure S3: *Gordonia polyisoprenivorans* strains in pangenomic comparison; Figure S4: Distribution of COG functional categories; Figure S5: Distribution of switched noncoding intergenic regions in the genome of *G. polyisoprenivorans* strain 135; Table S1: List of genes unique for the strain *G. polyisoprenivorans* 135; Table S2: Occurrence of genes (a) extradiol ring-cleavage dioxygenase and (b) aromatic ring-hydroxylating dioxygenase in *Actinobacteria* genomes.
