# Supplementary material for: Genomic, Phylogenetic and Physiological Characterization of the PAH-Degrading Strain Gordonia polyisoprenivorans 135
Source: Biology (Basel). 2024 May 13;13(5):339. doi: 10.3390/biology13050339 (PMC11117675; doi:10.3390/biology13050339)
Supplement: Supplementary file 1 [file biology-13-00339-s001.zip › biology-2981371-supplementary.pdf]

Figure S1. Colony appearance of the strain *Gordonia polyisoprenivorans* 135

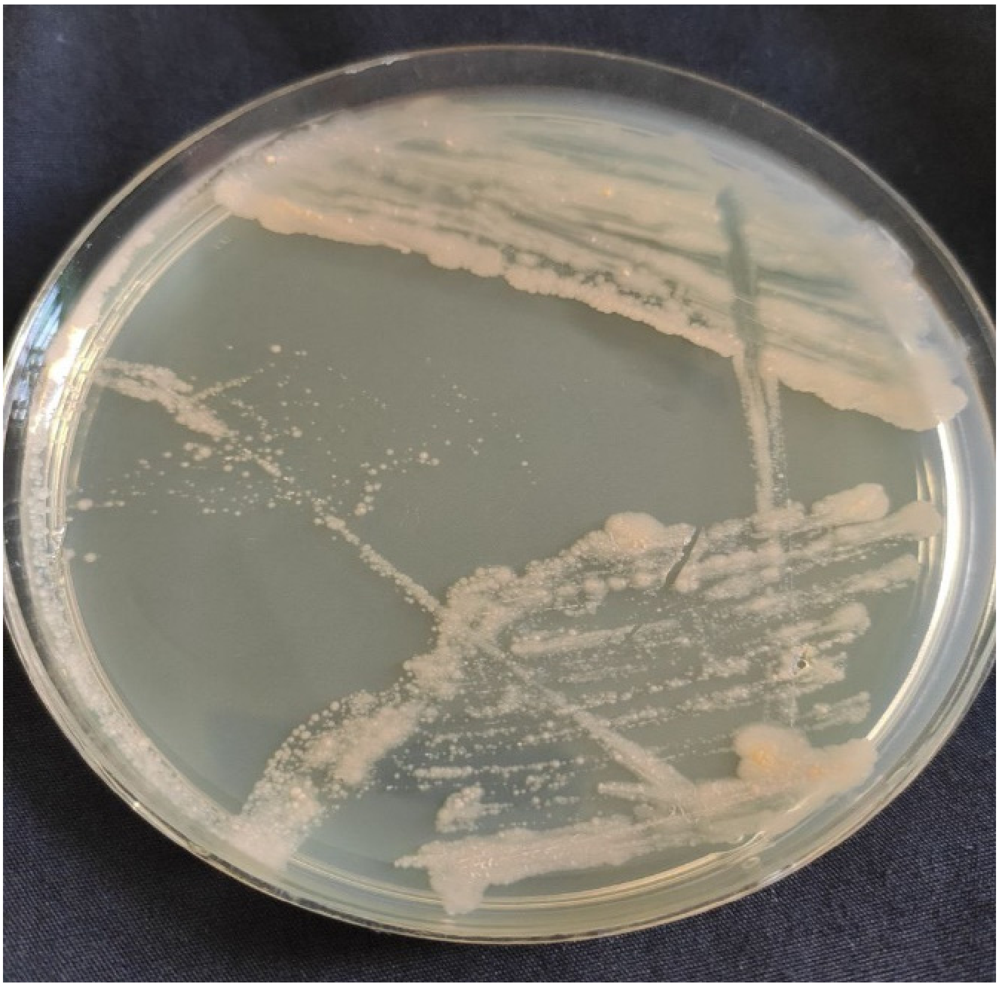

Figure S2. Circular genomic map of *G. polyisoprenivorans* 135 chromosome.

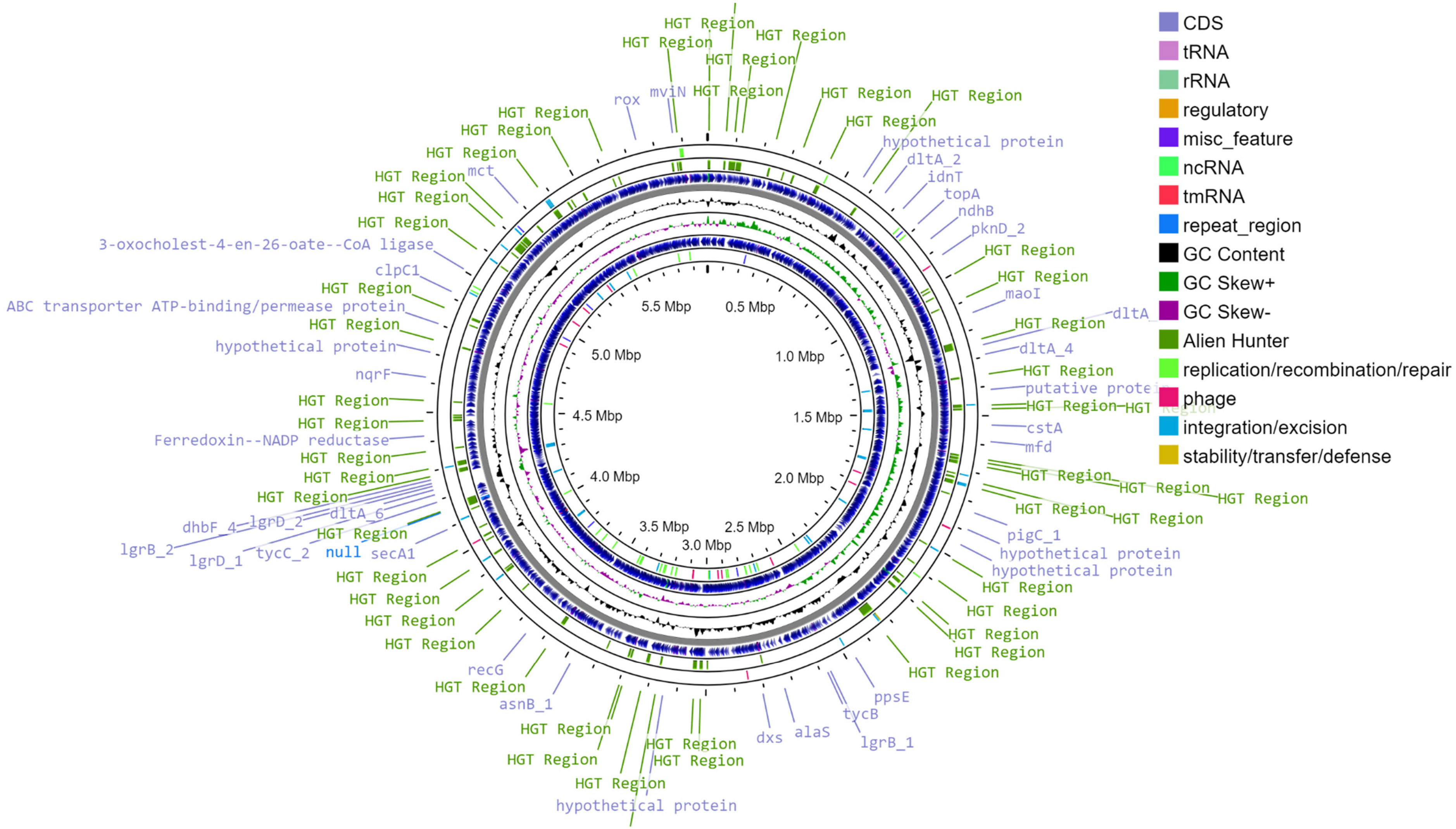

Figure S3. *Gordonia polyisoprenivorans* strains (a) 135, (b) C, (c) VH2 in pangenomic comparison. Tracks (from outside in): Forward genes (blue), Reverse genes (red), Core-genes (purple), Strain-specific genes (green), GC skew (black)

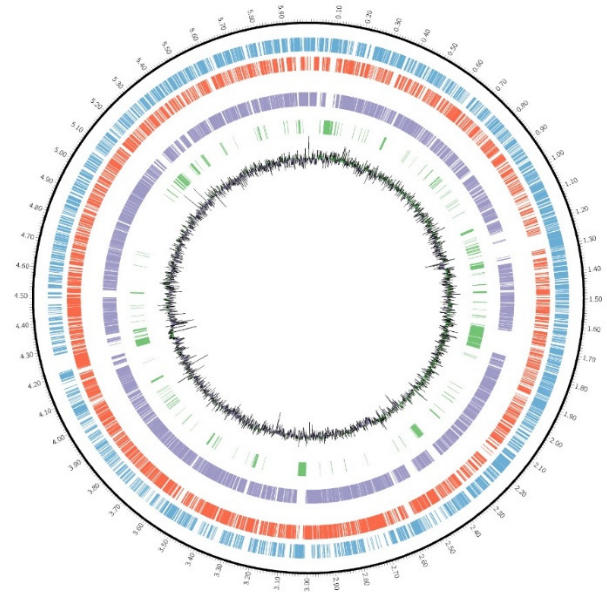

(a)

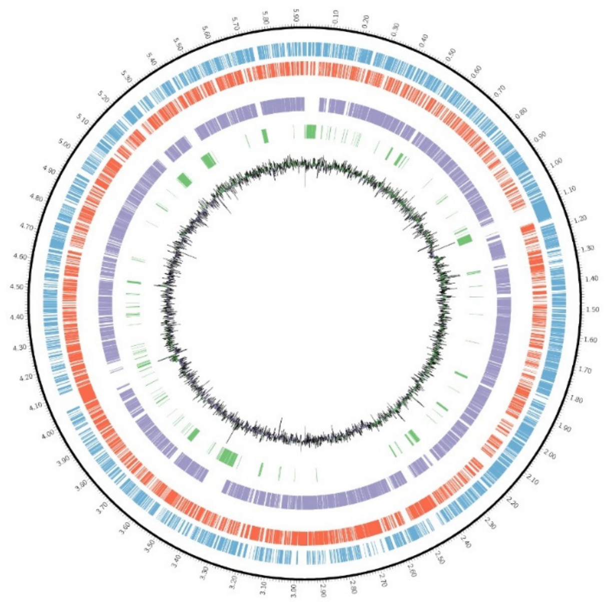

(b)

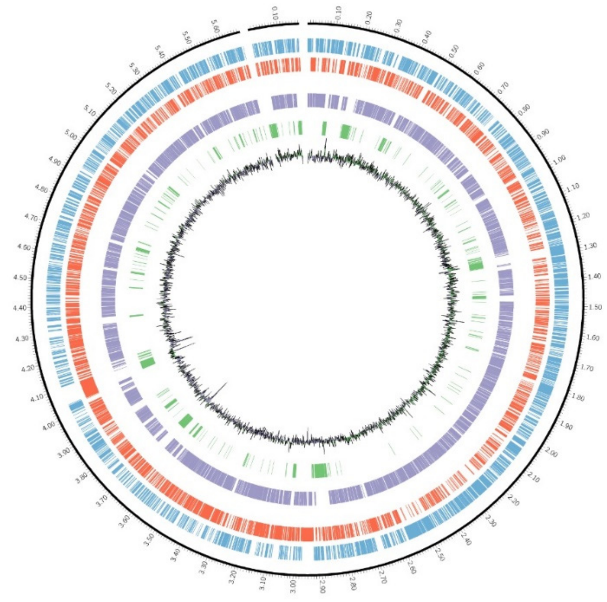

(c)

Figure S4. Distribution of COG functional categories.

INFORMATION STORAGE AND PROCESSING: [J] Translation, ribosomal structure and biogenesis, [A] RNA processing and modification, [K] Transcription, [L] Replication, recombination and repair, [B] Chromatin structure and dynamics

CELLULAR PROCESSES AND SIGNALING: [D] Cell cycle control, cell division, chromosome partitioning, [Y] Nuclear structure, [V] Defense mechanisms, [T] Signal transduction mechanisms, [M] Cell wall/membrane/envelope biogenesis, [N] Cell motility, [Z] Cytoskeleton, [W] Extracellular structures, [U] Intracellular trafficking, secretion, and vesicular transport, [O] Posttranslational modification, protein turnover, chaperones

METABOLISM: [C] Energy production and conversion, [G] Carbohydrate transport and metabolism, [E] Amino acid transport and metabolism, [F] Nucleotide transport and metabolism, [H] Coenzyme transport and metabolism, [I] Lipid transport and metabolism, [P] Inorganic ion transport and metabolism, [Q] Secondary metabolites biosynthesis, transport and catabolism

POORLY CHARACTERIZED: [R] General function prediction only, [S] Function unknown

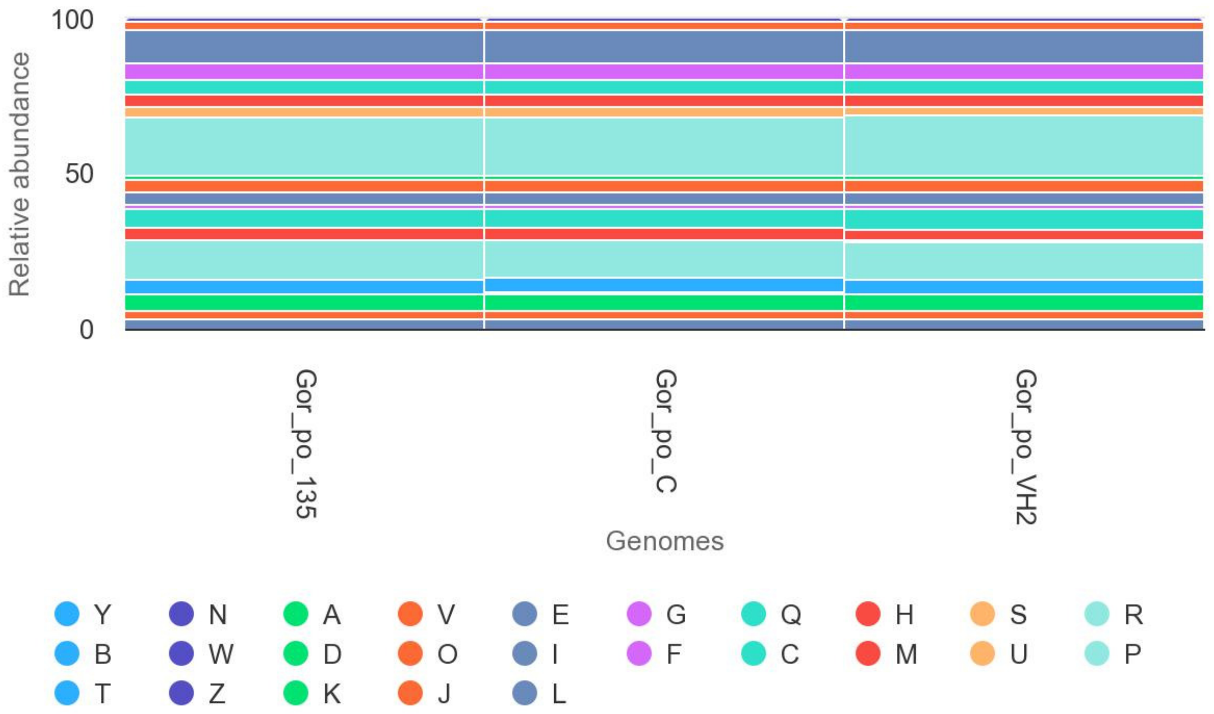

Table S1. List of genes unique for the strain *G. polyisoprenivorans* 135. COG category designations are given according to the legend in Figure S5. All hypothetical proteins and transposases have been removed from the list.

Cluster	Function	COG	COG categories
CLUSTER6226	MaoC/PaaZ C-terminal domain-containing protein	COG2030,COG3777	S,I
CLUSTER6223	cation-translocating P-type ATPase	COG0560,COG2216,COG0546,	E,R,P
CLUSTER6218	serine protease	#	#
CLUSTER6216	MarR family transcriptional regulator	COG1846	K
CLUSTER6215	MerR family transcriptional regulator	COG0789	K
CLUSTER6211	MarR family transcriptional regulator	COG0640,COG1321,COG1959,	K
CLUSTER6210	aromatic ring-hydroxylating dioxygenase subunit alpha	COG2146,COG4638	P
CLUSTER6209	AMP-binding protein	COG0365,COG1541,COG1021,	Q,H,I
CLUSTER6208	LuxR C-terminal-related transcriptional regulator	COG4566,COG2771,COG2203,	K,T
CLUSTER6190	DEAD/DEAH box helicase	COG1204,COG4581,COG4098,	K,L,V,R
CLUSTER6189	CHAT domain-containing protein	COG4995	S
CLUSTER6030	SRPBCC family protein	#	#
CLUSTER6028	metalloregulator ArsR/SmtB family transcription factor	COG3398,COG1654,COG1846,	K,S
CLUSTER6024	ATP-binding protein	COG2256,COG2074,COG0714,	G,R,T,E,O,L
CLUSTER6022	DHA2 family efflux MFS transporter permease subunit	COG0477,COG2814,COG2271	G
CLUSTER6017	MFS transporter	COG2211,COG2807,COG2814,	P,G
CLUSTER6016	non-heme iron oxygenase ferredoxin subunit	COG0723,COG2146,COG4638	C,P
CLUSTER6015	acyl-CoA dehydrogenase family protein	COG1960	I
CLUSTER6014	helix-turn-helix transcriptional regulator	COG1595,COG2909,COG2771,	T,K
CLUSTER6002	helix-turn-helix domain-containing protein	COG2207,COG4977	K
CLUSTER5887	VOC family protein	COG2514,COG0346	R,E
CLUSTER5885	MATE family efflux transporter	COG0728,COG0534	R,V
CLUSTER5871	amino acid adenylation domain-containing protein	COG1020,COG0236,COG0318,	I,H,Q
CLUSTER5866	SGNH/GDSL hydrolase family protein	COG1835	I
CLUSTER5861	TlpA disulfide reductase family protein	COG0526,COG3118	O
CLUSTER5854	LCP family protein	COG1316	K
CLUSTER5852	NADP-dependent alcohol dehydrogenase	COG0604,COG1062,COG1063,	R,C,E
CLUSTER5851	MspA family porin	#	#

CLUSTER5847	phosphatase PAP2 family protein	#	#
CLUSTER5846	DUF2834 domain-containing protein	#	#
CLUSTER5841	ABC transporter permease	COG1079,COG4177,COG4214,	G,R,E
CLUSTER5840	helix-turn-helix transcriptional regulator	COG2909,COG2522,COG4566,	K,T,R
CLUSTER5716	thiamine permease	COG1457	F
CLUSTER5708	fumarylacetoacetate hydrolase family protein	COG3970,COG0179	R,Q
CLUSTER5706	saccharopine dehydrogenase NADP-binding domain-containing protein	COG0300,COG3268,COG2910,	M,R,S,E
CLUSTER5698	MerR family transcriptional regulator	COG0789	K
CLUSTER5694	DNA/RNA non-specific endonuclease	#	#
CLUSTER5693	amino acid adenylation domain-containing protein	COG1020,COG0318,COG0365,	Q,I
CLUSTER5690	DNA cytosine-5--methyltransferase	COG0270	L
CLUSTER5689	acyltransferase	COG1835	I
CLUSTER5667	enoyl-CoA hydratase-related protein	COG0447,COG1024	H,I
CLUSTER5665	MspA family porin	#	#
CLUSTER5663	glycosyltransferase 87 family protein	#	#
CLUSTER5661	ATP-binding cassette domain-containing protein	COG4555,COG4148,COG1116,	Q,H,C,O,E,D,R,V,G,L,P
CLUSTER5656	LuxR family transcriptional regulator	COG2197,COG2771,COG4566,	K,T
CLUSTER5651	ATP-dependent Clp protease proteolytic subunit	COG0616,COG1030,COG0740	O
CLUSTER5533	hydantoinase/oxoprolinase family protein	COG0145	E
CLUSTER5531	AfsA-related hotdog domain-containing protein	#	#
CLUSTER5530	NADP/FAD-dependent oxidoreductase	COG0654,COG0644	C,H
CLUSTER5523	SDR family oxidoreductase	COG4221,COG3967,COG0300,	R,M,I
CLUSTER5517	beta-ketoacyl synthase N-terminal-like domain-containing protein	COG0304,COG3321,COG0331	Q,I
CLUSTER5511	amino acid adenylation domain-containing protein	COG1022,COG1021,COG1541,	I,Q,H
CLUSTER5502	very short patch repair endonuclease	COG3727	L
CLUSTER5487	acyl-CoA dehydrogenase family protein	COG1960	I
CLUSTER5486	AMP-binding protein	COG0365,COG1021,COG1022,	I,Q
CLUSTER5482	beta-16-N-acetylglucosaminyltransferase	#	#
CLUSTER5480	LacI family DNA-binding transcriptional regulator	COG1879,COG1609	G,K
CLUSTER5477	DUF1266 domain-containing protein	COG0515	R

CLUSTER5473	HNH endonuclease	COG1403	V
CLUSTER5355	transporter substrate-binding protein	COG0683	E
CLUSTER5353	AfsA-related hotdog domain-containing protein	#	#
CLUSTER5352	TetR/AcrR family transcriptional regulator	COG1309	K
CLUSTER5347	cyclase family protein	COG1878	R
CLUSTER5342	SDR family NADP-dependent oxidoreductase	COG1088,COG0236,COG1028	M,I
CLUSTER5338	antibiotic biosynthesis monooxygenase	COG1359	S
CLUSTER5337	condensation domain-containing protein	COG1020,COG0318,COG0365,	I,Q,H
CLUSTER5336	thiamine pyrophosphate-binding protein	COG0028	E
CLUSTER5321	Abi family protein	COG5520,COG4823	M,V
CLUSTER5320	carboxymuconolactone decarboxylase family protein	COG2128,COG0599	S
CLUSTER5316	Fis family transcriptional regulator	#	#
CLUSTER5312	SDR family oxidoreductase	COG1028,COG0623,COG0300,	I,R
CLUSTER5311	AMP-binding protein	COG1020,COG0318,COG1021,	I,Q,H
CLUSTER5307	O-antigen ligase family protein	COG3307	M
CLUSTER5303	helix-turn-helix domain-containing protein	COG3464	L
CLUSTER5182	helix-turn-helix domain-containing protein	COG3835,COG2508	T,K
CLUSTER5179	NADPH-dependent oxidoreductase	COG0655,COG0431,COG1182	I,R
CLUSTER5174	SDR family NADP-dependent oxidoreductase	COG3967,COG4221,COG0623,	R,M,I
CLUSTER5164	helix-turn-helix domain-containing protein	COG2207,COG2169,COG4977,	R,F,K
CLUSTER5163	trypsin-like peptidase domain-containing protein	COG0265,COG3480	O,T
CLUSTER5151	GNAT family N-acetyltransferase	COG0456,COG0454,COG1247,	K,J,M,R
CLUSTER5150	metal-sensitive transcriptional regulator	COG1937	S
CLUSTER5145	helix-turn-helix transcriptional regulator	COG3655	K
CLUSTER5136	alpha/beta hydrolase	COG0596,COG2267,COG1075	R,I
CLUSTER5135	NADP/FAD-dependent oxidoreductase	COG0654,COG0665,COG1053,	P,R,S,H,C,Q,E,O
CLUSTER5132	TetR/AcrR family transcriptional regulator	COG3226,COG1309	S,K
CLUSTER5129	alpha/beta fold hydrolase	COG0596	R
CLUSTER5127	type 1 glutamine amidotransferase domain-containing protein	COG0693,COG3155	Q,R
CLUSTER5010	ATP-binding protein	COG1106,COG4637,COG1195,	L,R
CLUSTER5008	amidohydrolase family protein	COG2159	R

CLUSTER5001	alpha/beta fold hydrolase	COG1506,COG1073,COG0412,	Q,R,E
CLUSTER4872	GAF domain-containing protein	COG2203,COG3290,COG3275,	K,T,H
CLUSTER4859	helix-turn-helix domain-containing protein	COG4977,COG1205,COG2207,	F,K,R
CLUSTER4855	NAD-dependent epimerase/dehydratase family protein	COG2910,COG1090,COG3268,	R,M,S,E
CLUSTER4851	helix-turn-helix domain-containing protein	COG1349	K
CLUSTER4843	ACR3 family arsenite efflux transporter	COG0798	P
CLUSTER4842	multicopper oxidase family protein	COG2132	Q
CLUSTER4831	DUF433 domain-containing protein	COG2442	S
CLUSTER4830	enoyl-CoA hydratase-related protein	COG0447,COG1024	H,I
CLUSTER4829	LLM class flavin-dependent oxidoreductase	COG2141	C
CLUSTER4828	MCE family protein	COG4372,COG0840,COG1511,	N,S,Q
CLUSTER4826	type II toxin-antitoxin system RelE/ParE family toxin	COG3549	R
CLUSTER4823	acyl-CoA/acyl-ACP dehydrogenase	COG1960	I
CLUSTER4806	allophanate hydrolase	COG0154	J
CLUSTER4671	YciI family protein	COG2350	S
CLUSTER4669	TetR/AcrR family transcriptional regulator	COG1395,COG1309	K
CLUSTER4659	AraC family transcriptional regulator	COG2169,COG2207,COG4977	F,K
CLUSTER4633	GNAT family protein	COG1670	J
CLUSTER4630	transporter substrate-binding protein	COG0683	E
CLUSTER4625	DUF5615 family PIN-like protein	COG4634,COG1656	S
CLUSTER4624	PaaX family transcriptional regulator C-terminal domain-containing protein	COG3327	K
CLUSTER4623	14-dihydroxy-2-naphthoyl-CoA synthase	COG1024,COG0616,COG0447	I,O,H
CLUSTER4622	MCE family protein	COG1463	Q
CLUSTER4621	HigA family addiction module antitoxin	COG5499,COG3093	K,R
CLUSTER4618	amino acid--[acyl-carrier-protein] ligase	COG4302,COG0172	E,J
CLUSTER4475	SulP family inorganic anion transporter	COG2233,COG0659,COG1366	P,F,T
CLUSTER4468	response regulator	COG2204,COG3437,COG2201,	N,T,K
CLUSTER4442	organomercurial lyase MerB	#	#
CLUSTER4424	4-hydroxy-2-oxovalerate aldolase	COG1038,COG5016,COG0119	E,C
CLUSTER4423	3-oxoacyl-ACP reductase FabG	COG0300,COG0451,COG0623,	R,M,I
CLUSTER4422	MCE family protein	COG1463,COG4192	Q,T

CLUSTER4420	GntR family transcriptional regulator	COG3355,COG1725,COG1167,	K
CLUSTER4263	EamA family transporter	COG2510,COG0697,COG5006	S,G,R
CLUSTER4260	GAP family protein	#	#
CLUSTER4254	serine hydrolase	COG1680	V
CLUSTER4245	ABC transporter permease	COG1172,COG4603,COG1079,	E,R,G
CLUSTER4236	LA2681 family HEPN domain-containing protein	#	#
CLUSTER4225	mercuryII reductase	COG0493,COG1251,COG0446,	C,H,R,E,O
CLUSTER4213	site-specific integrase	COG0582,COG4973	L
CLUSTER4205	acetaldehyde dehydrogenase acetylating	COG1063,COG0673,COG0287,	Q,R,E
CLUSTER4204	acetyl-CoA hydrolase/transferase C-terminal domain-containing protein	COG3051,COG0427,COG1349	K,C
CLUSTER4203	MlaD family protein	COG3008,COG1463	Q,R
CLUSTER4202	UvrD-helicase domain-containing protein	COG1074,COG3973,COG0210	L,R
CLUSTER4199	helix-turn-helix domain containing protein	COG1309	K
CLUSTER4198	3-oxoacyl-ACP reductase FabG	COG0300,COG0451,COG1028,	I,R,M
CLUSTER4190	DNA-binding protein	#	#
CLUSTER4041	fumarylacetoacetate hydrolase family protein	COG3970,COG0179	R,Q
CLUSTER4019	NADP/FAD-dependent oxidoreductase	COG0654	H
CLUSTER4009	heavy metal-responsive transcriptional regulator	COG0789,COG2452	L,K
CLUSTER4002	site-specific integrase	COG0582,COG4974	L
CLUSTER3996	DUF559 domain-containing protein	COG2852	S
CLUSTER3995	DUF262 domain-containing protein	COG1479	S
CLUSTER3852	TIGR02391 family protein	#	#
CLUSTER3828	M23 family metallopeptidase	COG4942,COG0739	D,M
CLUSTER3819	S8 family peptidase	COG1404	O
CLUSTER3817	MerR family transcriptional regulator	COG0789	K
CLUSTER3811	AMP-binding protein	COG1022,COG1021,COG1541,	Q,H,I
CLUSTER3810	FAD-dependent oxidoreductase	COG0644,COG0492,COG3634,	P,R,H,C,O
CLUSTER3809	MaoC family dehydratase N-terminal domain-containing protein	COG2030,COG3777	S,I
CLUSTER3808	FAD-dependent oxidoreductase	COG1249,COG3380,COG1252,	R,C,Q,E,S,O
CLUSTER3806	SGNH/GDSL hydrolase family protein	COG2755	E

CLUSTER3798	helix-turn-helix domain-containing protein	COG2169,COG2207,COG4977	F,K
CLUSTER3795	helix-turn-helix domain-containing protein	COG3464	L
CLUSTER3794	LysR family transcriptional regulator	COG0583,COG1321	K
CLUSTER3793	AAA family ATPase	COG0210,COG3973,COG1074	R,L
CLUSTER3652	undecaprenyl-diphosphate phosphatase	COG1968	V
CLUSTER3641	toxin glutamine deamidase domain-containing protein	#	#
CLUSTER3630	AraC family transcriptional regulator ligand-binding domain-containing protein	COG4977,COG2207,COG2169,	F,K
CLUSTER3626	c-type cytochrome biogenesis protein CcsB	COG4137,COG1138,COG0755	O,R
CLUSTER3609	hotdog domain-containing protein	COG2050,COG1607	I,Q
CLUSTER3608	extradiol ring-cleavage dioxygenase	#	#
CLUSTER3607	MaoC/PaaZ C-terminal domain-containing protein	COG2030,COG3777	S,I
CLUSTER3604	oligosaccharide repeat unit polymerase	#	#
CLUSTER3589	class A beta-lactamase-related serine hydrolase	COG2367,COG2027,COG0768,	M,V
CLUSTER3588	3-5 exonuclease	COG0210,COG3972,COG1074,	L,R
CLUSTER3447	DedA family protein	COG0586	S
CLUSTER3446	6-carboxytetrahydropterin synthase QueD	COG0720	H
CLUSTER3432	GMC family oxidoreductase	COG1053,COG1249,COG0644,	E,Q,C
CLUSTER3427	cytochrome c biogenesis protein ResB	COG1333	O
CLUSTER3424	DUF222 domain-containing protein	#	#
CLUSTER3417	SDR family NADP-dependent oxidoreductase	COG0300,COG1028,COG0623,	R,M,I
CLUSTER3416	IclR family transcriptional regulator	COG2512,COG1522,COG4190,	K,S
CLUSTER3415	zinc-binding dehydrogenase	COG1064,COG2130,COG0604,	R,C,E
CLUSTER3414	CoA ester lyase	COG2301	G
CLUSTER3410	DUF4012 domain-containing protein	#	#
CLUSTER3402	FUSC family protein	COG1289,COG4129	S
CLUSTER3397	penicillin-binding transpeptidase domain-containing protein	COG0768,COG4953,COG5009,	M
CLUSTER3396	DEAD/DEAH box helicase	COG1201,COG1061,COG0513,	K,L,R
CLUSTER3256	ATP-binding cassette domain-containing protein	COG4152,COG1117,COG4586,	F,P,V,G,R,L,N,D,Q,H,C,O,E
CLUSTER3255	7-carboxy-7-deazaguanine synthase	COG1180,COG2896,COG0602	H,O
CLUSTER3239	FAD-dependent oxidoreductase	COG4529,COG0492,COG1232,	H,E,S,O

CLUSTER3237	cytochrome c biogenesis protein ResB	COG1333	O
CLUSTER3231	ImmA/IrrE family metallo-endopeptidase	COG2856	E
CLUSTER3226	PaaX family transcriptional regulator C-terminal domain-containing protein	COG3327	K
CLUSTER3225	amidohydrolase family protein	COG2159	R
CLUSTER3224	hotdog fold thioesterase	COG2050	Q
CLUSTER3223	MaoC family dehydratase	COG2030	I
CLUSTER3221	glycosyltransferase family 4 protein	COG0438	M
CLUSTER3214	MarR family transcriptional regulator	COG1378,COG1846,COG1510,	K,P,S
CLUSTER3210	restriction endonuclease subunit M	#	#
CLUSTER3073	ABC transporter permease subunit	COG1173,COG4171,COG4239	V,R,E
CLUSTER3058	DUF4873 domain-containing protein	#	#
CLUSTER3056	twin-arginine translocation signal domain-containing protein	COG4263	C
CLUSTER3048	2-hydroxyacyl-CoA dehydratase family protein	COG1775	E
CLUSTER3047	NADP/FAD-dependent oxidoreductase	COG1232,COG0654	H
CLUSTER3046	AMP-binding protein	COG0318,COG1020,COG1022,	I,Q
CLUSTER3045	AMP-binding protein	COG0365,COG1021,COG1022,	I,Q
CLUSTER3043	DapH/DapD/GlmU-related protein	COG1207,COG0110,COG1044,	E,M,R
CLUSTER3038	AraC family transcriptional regulator N-terminal domain-containing protein	COG2207,COG4977,COG4753	K,T
CLUSTER3036	MFS transporter	COG2211,COG2814,COG0477,	G
CLUSTER3025	DEAD/DEAH box helicase	COG4096,COG0553,COG1061,	K,L,R,V
CLUSTER2912	TetR/AcrR family transcriptional regulator	COG1309	K
CLUSTER2905	ABC transporter permease	COG1173,COG4168,COG0601	V,E
CLUSTER2898	polysaccharide pyruvyl transferase family protein	COG5039	G
CLUSTER2895	alpha/beta hydrolase	COG0596,COG3319,COG2267,	I,E,Q,R
CLUSTER2890	diiron oxygenase	#	#
CLUSTER2884	multiubiquitin domain-containing protein	#	#
CLUSTER2882	DUF4345 domain-containing protein	#	#
CLUSTER2881	2-hydroxyacyl-CoA dehydratase family protein	COG2441,COG1775	E,C
CLUSTER2880	alpha/beta hydrolase	COG1506,COG1647,COG0596,	R,I,E

CLUSTER2879	aldehyde dehydrogenase	COG1012,COG4230	C
CLUSTER2876	endo-13-alpha-glucanase family glycosylhydrolase	#	#
CLUSTER2875	polysaccharide biosynthesis tyrosine autokinase	COG0541,COG0003,COG3944,	P,U,D,M,T
CLUSTER2864	Lrp/AsnC family transcriptional regulator	COG1522	K
CLUSTER2862	DUF1998 domain-containing protein	#	#
CLUSTER2744	TetR/AcrR family transcriptional regulator	COG1309,COG1193	L,K
CLUSTER2743	ABC transporter substrate-binding protein	COG4533,COG4166,COG0747	E,R
CLUSTER2742	PfkB family carbohydrate kinase	COG3613,COG0524,COG1105	F,G
CLUSTER2738	NADP-dependent oxidoreductase	COG1250,COG0362,COG1893,	H,C,M,E,R,G,S,I,P
CLUSTER2729	TetR/AcrR family transcriptional regulator	COG1309	K
CLUSTER2728	apolipoprotein N-acyltransferase	COG0388,COG0815	R,M
CLUSTER2726	beta-ketoacyl synthase N-terminal-like domain-containing protein	COG0304,COG3321	Q,I
CLUSTER2724	TetR/AcrR family transcriptional regulator	COG1309	K
CLUSTER2722	acyl-CoA dehydratase activase	COG1940,COG0068,COG1070,	G,I,O,K
CLUSTER2721	AMP-binding protein	COG1022,COG0365,COG1021,	I,Q
CLUSTER2720	LuxR C-terminal-related transcriptional regulator	COG2197,COG4566,COG2771,	K,T
CLUSTER2717	glycosyltransferase	COG1215,COG1216	M,R
CLUSTER2705	helicase-related protein	#	#
CLUSTER2594	putative quinol monooxygenase	COG1359	S
CLUSTER2592	TauD/TfdA family dioxygenase	COG2175	Q
CLUSTER2591	7-cyano-7-deazaguanine synthase	COG0367,COG0603	E,R
CLUSTER2584	type IV toxin-antitoxin system AbiEi family antitoxin domain-containing protein	COG2852	S
CLUSTER2580	fluoride efflux transporter CrcB	COG0239	D
CLUSTER2567	ThiF family adenylyltransferase	COG1179,COG0476	H
CLUSTER2561	SDR family NADP-dependent oxidoreductase	COG3967,COG4221,COG0169,	R,M,E,I
CLUSTER2560	LuxR C-terminal-related transcriptional regulator	COG1595,COG1522,COG2909,	K,T
CLUSTER2555	GDP-mannose 46-dehydratase	COG0702,COG0451,COG1088,	M
CLUSTER2552	hydrolase	COG0388,COG0815	R,M
CLUSTER2548	AAA family ATPase	COG0210,COG1074	L
CLUSTER2428	NADP/FAD-dependent oxidoreductase	COG1233,COG1148,COG1249,	P,S,R,E,O,C,H,Q

CLUSTER2426	ornithine cyclodeaminase family protein	COG2423	E
CLUSTER2425	nitroreductase family protein	#	#
CLUSTER2418	glycosyltransferase family 2 protein	COG0463,COG1215,COG1216	R,M
CLUSTER2415	SRPBCC domain-containing protein	COG3832	S
CLUSTER2411	cytochrome c biogenesis protein CcdA	COG4232,COG2194,COG0785	R,O
CLUSTER2408	MerR family transcriptional regulator	COG0789	K
CLUSTER2407	Fis family transcriptional regulator	#	#
CLUSTER2403	tyrosine-protein phosphatase	COG2365,COG5599,COG2453	T
CLUSTER2402	acyl-CoA/acyl-ACP dehydrogenase	COG1960	I
CLUSTER2401	2Fe-2S iron-sulfur cluster-binding protein	COG0633,COG3894,COG2871	C,R
CLUSTER2399	TetR/AcrR family transcriptional regulator	COG1309,COG2207	K
CLUSTER2398	acyl-CoA/acyl-ACP dehydrogenase	COG1960	I
CLUSTER2397	GDP-L-fucose synthase	COG1088,COG1087,COG0451,	M
CLUSTER2393	ester cyclase	COG5485	R
CLUSTER2383	very short patch repair endonuclease	COG3727,COG2852	L,S
CLUSTER2258	helix-turn-helix domain-containing protein	#	#
CLUSTER2256	LysR family transcriptional regulator	COG2522,COG0583	R,K
CLUSTER2254	alanine racemase	COG1166,COG0019,COG0787	M,E
CLUSTER2241	ATP-binding cassette domain-containing protein	COG4161,COG3845,COG2401,	V,G,R,L,P,H,C,Q,T,E,O,D
CLUSTER2230	TlpA disulfide reductase family protein	COG0526,COG3118	O
CLUSTER2224	helix-turn-helix transcriptional regulator	COG3655	K
CLUSTER2216	PaaI family thioesterase	COG2050	Q
CLUSTER2215	MarR family transcriptional regulator	COG1846,COG1321	K
CLUSTER2214	cytochrome P450	COG2124	Q
CLUSTER2209	Rrf2 family transcriptional regulator	COG1959,COG1725	K
CLUSTER2208	YdcF family protein	COG1434	S
CLUSTER2207	sialate O-acetylesterase	COG1506,COG0412	E,Q
CLUSTER2206	histidinol dehydrogenase	COG0141	E
CLUSTER2196	phosphoribosyltransferase	COG0503,COG1040,COG0461,	F,R
CLUSTER2068	FAD-dependent monooxygenase	COG0644,COG1148,COG0493,	S,E,H,R,C,Q
CLUSTER2067	cysteine synthase family protein	COG1171,COG0031,COG0498	E
CLUSTER2035	tyrosine-type recombinase/integrase	COG4973,COG0582,COG4974	L

CLUSTER2026	CoA transferase	COG1804	C
CLUSTER2025	FAD-dependent oxidoreductase	COG3634,COG0492,COG0569,	P,C,R,E,O
CLUSTER2020	acyl-CoA/acyl-ACP dehydrogenase	COG1960	I
CLUSTER2019	class I SAM-dependent methyltransferase	COG4106,COG2227,COG0500	H,R,Q
CLUSTER2016	ABC transporter substrate-binding protein	COG1879,COG4213	G
CLUSTER2002	DNA-processing protein DprA	COG0758	L
CLUSTER1992	ATP-binding protein	COG3899	R
CLUSTER1982	linear amide C-N hydrolase	COG3049	M
CLUSTER1969	metalloregulator ArsR/SmtB family transcription factor	COG2345,COG3355,COG1846,	K,S
CLUSTER1968	heavy metal translocating P-type ATPase	COG4087,COG2217,COG1778,	E,R,G,P
CLUSTER1964	HAD-IA family hydrolase	COG0546,COG0637	R
CLUSTER1963	tyrosine-type recombinase/integrase	COG4974,COG0582,COG4973	L
CLUSTER1951	nuclear transport factor 2 family protein	#	#
CLUSTER1944	histidinol dehydrogenase	COG0141	E
CLUSTER1941	MarR family transcriptional regulator	COG1733,COG1846	K
CLUSTER1819	amidohydrolase family protein	COG3964,COG0044,COG3653,	R,G,Q,P,F
CLUSTER1812	helix-turn-helix domain-containing protein	#	#
CLUSTER1796	ATP-binding cassette domain-containing protein	COG4988,COG4555,COG0410,	O,E,Q,C,V,R,G,P
CLUSTER1781	NADP-binding domain-containing protein	COG2072,COG1249,COG1233,	O,E,S,Q,R,C,P
CLUSTER1780	YdhK family protein	#	#
CLUSTER1768	phosphotransferase family protein	COG2334,COG3173	R
CLUSTER1767	NDMA-dependent alcohol dehydrogenase	COG0604,COG0169,COG1063,	R,C,E
CLUSTER1761	SDR family NADP-dependent oxidoreductase	COG0300,COG0623,COG0451,	I,R,M
CLUSTER1756	DUF202 domain-containing protein	COG2149	S
CLUSTER1752	cold shock domain-containing protein	#	#
CLUSTER1751	oxidoreductase C-terminal domain-containing protein	#	#
CLUSTER1637	MarR family winged helix-turn-helix transcriptional regulator	COG1846	K
CLUSTER1619	YhgE/Pip domain-containing protein	COG1511,COG1463,COG0842,	N,S,Q,G,V
CLUSTER1607	NADP/FAD-dependent oxidoreductase	COG0438,COG1252,COG2072,	O,C,H,Q,M,S,R,P
CLUSTER1606	NADP/FAD-dependent oxidoreductase	COG2072,COG0644,COG0492,	H,C,Q,E,O,P
CLUSTER1592	AMP-binding protein	COG1022,COG1021,COG1541,	Q,H,I

CLUSTER1591	enoyl-CoA hydratase-related protein	COG1024,COG0447	H,I
CLUSTER1590	ABC transporter permease	COG0767	Q
CLUSTER1589	LysR family substrate-binding domain-containing protein	COG0583	K
CLUSTER1587	holo-ACP synthase	COG0736	I
CLUSTER1583	aldolase/citrate lyase family protein	COG3836,COG2301	G
CLUSTER1570	AraC family transcriptional regulator	COG4753,COG2169,COG2207,	K,F,T
CLUSTER1441	DUF5593 domain-containing protein	#	#
CLUSTER1434	enoyl-CoA hydratase/isomerase family protein	COG0447,COG1024	H,I
CLUSTER1416	MFS transporter	COG2814,COG0477,COG2807,	G,P
CLUSTER1415	heavy metal translocating P-type ATPase	COG0474,COG0561,COG2608,	R,E,P
CLUSTER1402	acyl-CoA/acyl-ACP dehydrogenase	COG1960	I
CLUSTER1401	enoyl-CoA hydratase-related protein	COG1024,COG0447	H,I
CLUSTER1400	ABC transporter permease	COG0767	Q
CLUSTER1398	LysR family transcriptional regulator	COG0583	K
CLUSTER1389	acyltransferase	COG1835	I
CLUSTER1387	WhiB family transcriptional regulator	#	#
CLUSTER1384	DUF1942 domain-containing protein	#	#
CLUSTER1251	Lrp/AsnC family transcriptional regulator	COG1522	K
CLUSTER1248	EthD domain-containing protein	#	#
CLUSTER1227	low molecular weight phosphatase family protein	COG0394	T
CLUSTER1226	heavy metal-associated domain-containing protein	COG2217,COG2608	P
CLUSTER1214	zinc ribbon domain-containing protein	#	#
CLUSTER1210	AMP-binding protein	COG1022,COG1021,COG0365,	I,Q
CLUSTER1209	enoyl-CoA hydratase-related protein	COG0447,COG1024	I,H
CLUSTER1208	MCE family protein	COG1463	Q
CLUSTER1203	DUF1839 family protein	#	#
CLUSTER1192	GntR family transcriptional regulator	COG1167,COG1725,COG2186,	K
CLUSTER1066	DUF4209 domain-containing protein	#	#
CLUSTER1065	ester cyclase	COG3631,COG5485	R
CLUSTER1058	LuxR C-terminal-related transcriptional regulator	COG2197,COG4566,COG2771,	K,T
CLUSTER1055	AraC family transcriptional regulator	COG2207,COG4977	K

CLUSTER1041	metalloregulator ArsR/SmtB family transcription factor	COG0640,COG4189,COG0394	K,T
CLUSTER1022	NUDIX domain-containing protein	#	#
CLUSTER1021	FadR/GntR family transcriptional regulator	COG1725,COG2186,COG1802,	K
CLUSTER1020	MlaD family protein	COG1463	Q
CLUSTER1015	acyl carrier protein	COG0236	I
CLUSTER1013	23-butanediol dehydrogenase	COG0604,COG0169,COG2072,	E,R,C,P
CLUSTER1006	DUF2742 domain-containing protein	#	#
CLUSTER1003	cysteine hydrolase	COG1335,COG1535	Q
CLUSTER0995	fumarylacetoacetate hydrolase family protein	COG3971	Q
CLUSTER0994	thiolase family protein	COG0183	I
CLUSTER0990	TetR/AcrR family transcriptional regulator C-terminal ligand-binding domain-containing protein	#	#
CLUSTER0989	sugar transferase	COG2148,COG1086	M
CLUSTER0988	phosphotransferase	COG1718,COG2334,COG3173	R,T
CLUSTER0977	helix-turn-helix transcriptional regulator	#	#
CLUSTER0972	PD-D/EXK nuclease family protein	#	#
CLUSTER0774	permease	COG0701	R
CLUSTER0766	DNA cytosine methyltransferase	COG0270	L
CLUSTER0757	helix-turn-helix domain containing protein	COG1309	K
CLUSTER0756	aromatic-ring-hydroxylating dioxygenase subunit beta	COG5517	Q
CLUSTER0755	SDR family NADP-dependent oxidoreductase	COG4221,COG3967,COG0300,	M,R,I
CLUSTER0748	APC family permease	COG1113,COG0531	E
CLUSTER0736	site-specific integrase	COG4973,COG4974,COG0582	L
CLUSTER0548	helix-turn-helix domain-containing protein	COG5484,COG3415	L,S
CLUSTER0523	class I SAM-dependent methyltransferase	COG0703,COG5624	E,K
CLUSTER0495	fatty acid--CoA ligase family protein	COG0318,COG1020,COG1022,	Q,H,I
CLUSTER0494	SDR family oxidoreductase	COG3967,COG4982,COG4221,	E,I,R,M
CLUSTER0493	acyl-CoA/acyl-ACP dehydrogenase	COG1960	I
CLUSTER0492	cytochrome P450	COG2124	Q
CLUSTER0485	aminotransferase class III-fold pyridoxal phosphate-dependent enzyme	COG0160,COG4992,COG0161,	E,H
CLUSTER0482	helix-turn-helix domain-containing protein	COG1733	K

CLUSTER0243	SDR family oxidoreductase	COG0300,COG1028,COG0623,	M,R,I
CLUSTER0238	signal peptidase II	COG0597	M
CLUSTER0218	TetR family transcriptional regulator	COG1309	K
CLUSTER0217	dienelactone hydrolase family protein	COG0412	Q
CLUSTER0216	acyl-CoA dehydrogenase family protein	COG1960	I
CLUSTER0215	aldehyde dehydrogenase	COG1012,COG4230	C
CLUSTER0209	glycosyltransferase	COG0763,COG1819,COG0297,	G,M
CLUSTER0205	zinc-dependent alcohol dehydrogenase family protein	COG1062,COG1063,COG0604,	E,R,C
CLUSTER0193	DNA cytosine methyltransferase	COG0270	L

Figure S5. Distribution of switched noncoding intergenic regions in the genome of *G. polyisoprenivorans* strain 135. NcIGRs have numbers corresponding to the numbers of genes lying downstream of these regions. The color scheme is as follows: yellow indicates IGRs lying between forward co-oriented genes; green indicates IGRs lying between reverse co-oriented genes; cyan indicates IGRs whose gene environment is transcribed divergently (in different directions); gray indicates IGRs whose gene environment is transcribed convergently (toward).

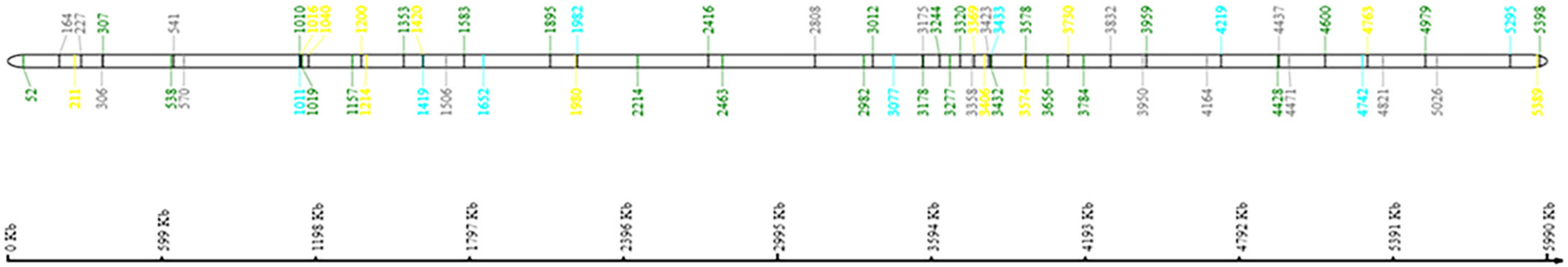

Schematic representation of the chromosome of *G. polyisoprenivorans* 135

Table S2. Occurrence of genes (a) extradiol ring-cleavage dioxygenase (WCB38962.1) and (b) aromatic ring-hydroxylating dioxygenase subunit alpha (WCB38965.1) in *Actinobacteria* genomes. The percent identity threshold was taken > 69%. Here, percent identity is to be understood as the percent of nucleotides that are identical between the query and database sequences. The databases nr/nt and WGS were used for analysis.

	**Genbank acc. number**	**Strain name**	**Query cover, %**	**Percent identity, %**
(a)	CP045809.1	*Gordonia pseudamarae* strain CON9 chromosome	99	79.25
	CP045806.1	*Gordonia pseudamarae* strain BEN371 chromosome	99	79.25
	CP053928.1	*Nocardioides* sp. WS12 chromosome	99	76.40
	CP089312.1	*Streptomyces* sp. GQFP chromosome	98	72.91
	CP015203.1	*Rhodococcus* sp. 008 plasmid pR8L1	98	72.54
	CP096567.1	*Rhodococcus qingshengii* JCM 15477 strain djl-6 plasmid pdjl-6-4	98	72.54
	CP054208.1	*Rhodococcus qingshengii* strain CX-1 plasmid unnamed1	98	72.54
	CP044283.1	*Rhodococcus erythropolis* strain X5 plasmid pRhX5	98	72.11
	CP016353.1	*Prauserella marina* strain DSM 45268 chromosome	99	69.74
	CP053564.1	*Pseudonocardia broussonetiae* strain Gen 01 chromosome	98	69.97
	CP012185.1	*Pseudonocardia* sp. EC080619-01 plasmid pBCI1-2	98	69.88
	CP012182.1	*Pseudonocardia* sp. EC080610-09 plasmid pBCI2-1	98	69.88
	CP072203.1	*Gordonia polyisoprenivorans* strain R9 chromosome	98	69.33
(b)	CP045809.1	*Gordonia pseudamarae* strain CON9 chromosome	97	80.76
	CP045806.1	*Gordonia pseudamarae* strain BEN371 chromosome	97	80.76
	CP053928.1	*Nocardioides* sp. WS12 chromosome	92	77.86
	CP089312.1	*Streptomyces* sp. GQFP chromosome	95	74.20
	CP044283.1	*Rhodococcus erythropolis* strain X5 plasmid pRhX5	92	74.06
	CP012185.1	*Pseudonocardia* sp. EC080619-01 plasmid pBCI1-2	95	72.95
	CP012182.1	*Pseudonocardia* sp. EC080610-09 plasmid pBCI2-1	95	72.95
	CP016353.1	*Prauserella marina* strain DSM 45268 chromosome	93	72.76
	CP015203.1	*Rhodococcus* sp. 008 plasmid pR8L1	92	72.21
	CP096567.1	*Rhodococcus qingshengii* JCM 15477 strain djl-6 plasmid pdjl-6-4	92	72.21
	CP054208.1	*Rhodococcus qingshengii* strain CX-1 plasmid unnamed1	92	72.21
	CP031414.1	*Mycolicibacterium neoaurum* strain HGMS2 chromosome	94	70.82
	CP011022.1	*Mycolicibacterium neoaurum* strain NRRL B-3805 chromosome	94	70.82
	CP006936.2	*Mycobacterium neoaurum* VKM Ac-1815D	94	70.82
	CP072203.1	*Gordonia polyisoprenivorans* strain R9 chromosome	94	70.41
